# High expression of folate cycle enzyme MTHFD1L correlates with poor prognosis and increased proliferation and migration in colorectal cancer

**DOI:** 10.7150/jca.35014

**Published:** 2020-04-25

**Authors:** Zhongyun He, Xia Wang, Huizhong Zhang, Baoxia Liang, Jinling Zhang, Zhenfeng Zhang, Yi Yang

**Affiliations:** 1 Department of Pathology, The Second Affiliated Hospital of Guangzhou Medical University, Guangzhou, PR, China.; 2 Department of Radiology, The Second Affiliated Hospital of Guangzhou Medical University, Guangzhou, PR, China.; 3 Department of Pathology, Sun Yat-sen University Cancer Center, Guangzhou, PR, China.; 4 Boba Evergrande International Hospital, Qionghai, PR, China.

**Keywords:** colorectal cancer, MTHFD1L, prognosis, migration, proliferation

## Abstract

**Aims:** To investigate the expression and clinical significance of methylenetetrahydrofolate dehydrogenase 1-like (MTHFD1L) in colorectal cancer (CRC) and its effect on CRC cells proliferation and migration.

**Methods:** 59 fresh CRC tissue samples and matched normal tissues, 176 archive CRC tissue samples and 8 CRC cell lines were tested MTHFD1L by western blot and immunohistochemistry, respectively. The relationship between MTHFD1L expression, clinical significance and prognosis was analyzed by chi-square test and survival analysis. MTT assay, plate clonal formation assay and scratch assay were used to verify the effect of MTHFD1L on the proliferation and migration in CRC cell lines.

**Results:** The results showed that the protein level of MTHFD1L in CRC was significantly higher than that in adjacent normal tissues (p<0.01). The expression of MTHFD1L in CRC was positively correlated with the degree of tumor differentiation, TNM classification, tumor invasion, lymph node metastasis, and distant metastasis. Survival analysis showed that CRC patients with high MTHFD1L expression had a lower 5-year survival rate and the expression of MTHFD1L was an independent adverse factor for the CRC prognosis (p<0.05). Down-regulation of MTHFD1L inhibited the proliferation and migration of DLD-1 and HCT116 CRC cell lines.

**Conclusion:** These findings reveal that MTHFD1L is highly expressive in CRC and associated with poor prognosis, and MTHDF1L can increase colorectal cancer cell proliferation and migration. Therefore, MTHFD1L may serve as a predictor and a potential therapeutic target for CRC.

## Introduction

According to the world health organization international cancer research center (IARC), the new morbidity and mortality rate of CRC ranks the third in the world^1^. Nowadays, surgery is the most important treatment for CRC, but tumor recurrence and metastasis after surgery are the main causes of death in patients with CRC. However, CRC lacks of new specific prognostic biomarkers and therapeutic targets. Therefore, it is urgent to search a key marker for CRC prognosis and therapy.

**Figure 1:**
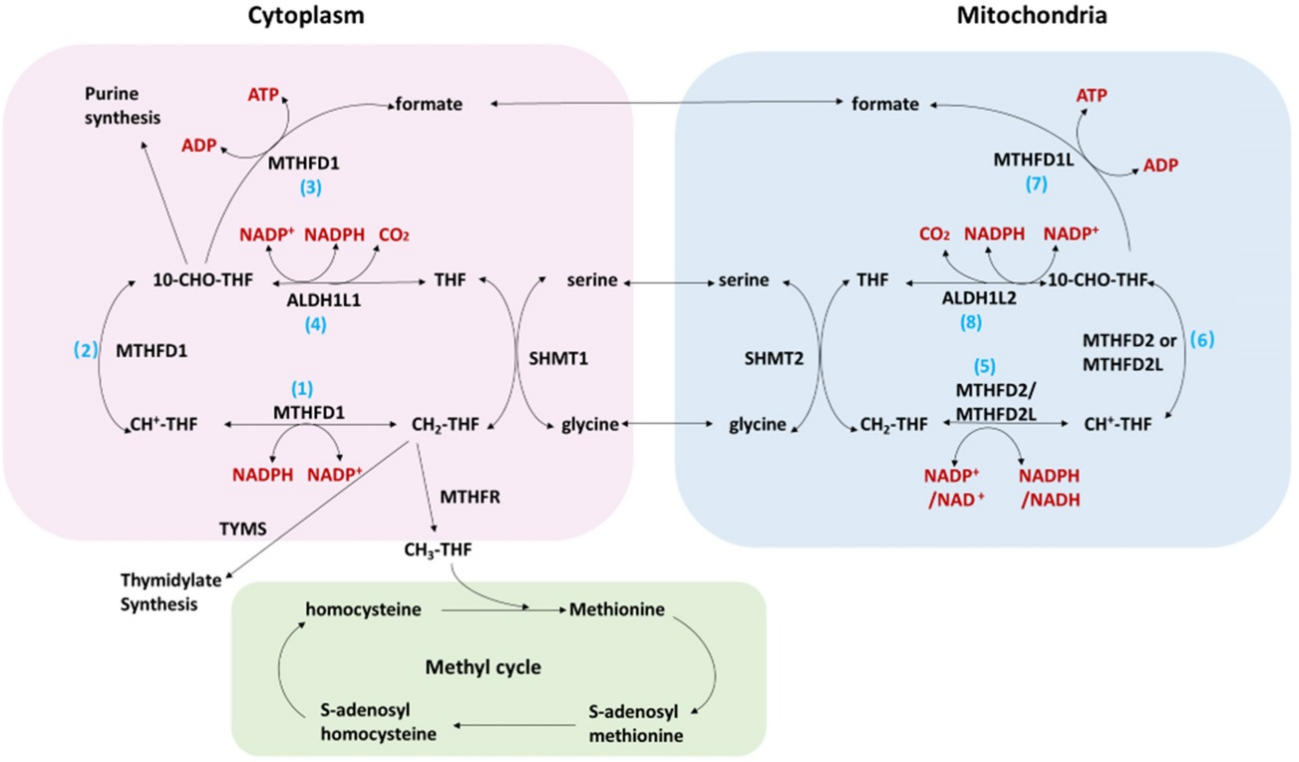
The folate cycle in cancer.

Folate cycling plays an important role in cellular physiological and pathological processes. The transport of one-carbon units (such as serine, glycine and formate) on the mitochondrial membrane enables the cytoplasm and mitochondrial compartment to be connected (figure 1). The connection between mitochondria and cytoplasm supports the unidirectional flow of one-carbon units from serine to formate, purine, pyrimidine, methionine, and NADPH. Studies^2^ have shown that a majority of one-carbon units (>75%) entering the cytoplasmic methionine cycle were derived from the mitochondria, which further confirms the connection between the two compartment. The one-carbon unit is exported to the cytoplasm in the form of formates and in the circulation of the two compartments produces many metabolites necessary for cell growth. The cytoplasmic folic acid cycling intermediate 10-CHO-THF can be used for purine synthesis, while CH2-THF can be used for the synthesis of pyrimidine. Methyltetrahydrofolate reductase (MTHFR) converts CH2-THF to CH3-THF which is connected to the methionine cycle. The one-carbon unit from CH3-THF is donated to the donor of S-adenosylmethionine for histone and DNA methylation. In addition, the folate cycle is an important source of NADPH^3^ which is a major cellular antioxidant. MTHFD1L is the enzyme catalyzing the last step of the mitochondrial compartment generating formate and could subsequently enter the cytoplasmic compartment. MTHFD1L therefore plays critical roles in folate cycle maintenance 4.

Folic acid metabolism is closely related to the development of various tumors^5^. The folate cycle is a complex metabolic network that controls nucleotide biosynthesis, methylation and redox maintenance. Among them, MTHFD2, SHMT2 and MTHFD1L are the core enzymes of the folate pathway^5^. Two key enzymes in the folate cycle, SHMT2 and MTHFD2, are associated with the occurrence and development of breast cancer, colorectal cancer and intrahepatic cholangiocarcinoma^6^-^9^. Methylenetetrahydrofolate dehydrogenase (NADP + dependent) 1 like (MTHFD1L) is the key enzyme in the last step of the folate cycle in the mitochondrial chamber and plays a key role in the folate pathway^2^. Previous studies have focused on its role in neural tube defects^10^, coronary artery disease^11^, depression^12^ and Alzheimer's disease^13^. Presently, several studies have shown that MTHFD1L played an important role in the development of liver cancer and esophageal cancer^14^, ^15^. However, there is no relative publications have proposed the role of MTHFD1L in CRC. In this study we firstly discussed the potential roles of MTHFD1L in CRC.

## Material and Methods

### Patients and tissue preparation

59 cases of fresh CRC tissues and corresponding normal tissues with 5cm distance far with the tumor were selected at the Second Affiliated Hospital of Guangzhou Medical University (Guangzhou, China) from January 2016 to December 2017.Samples were obtained in vitro within 20 min, and then the samples were immersed in liquid nitrogen for Western blot analysis. Another 176 cases of achive colon tumor and corresponding para-cancerous normal tissue blocks were chosen from January 2005 to December 2007 in the Second Affiliated Hospital of Guangzhou Medical University (Guangzhou, China), which were used for immunohistochemical detection. All of the cases were diagnosed as colorectal cancer by three experienced pathologists' double-blind observation. All cases were approved by the Research Ethics Committee of the Second Affiliated Hospital of Guangzhou Medical University, and all patients provided written informed consent.

### Cell culture

Human CRC cell lines HCT116, LS174T, LOVO, DLD-1, SW620, SW480, HCT-8, HCT-8, HT-29 and human intestinal mucosal epithelial cells NCM460 were purchased from the Shanghai cell bank of the Chinese academy of sciences. All the cells were cultured in RPMI 1640(Gibco) medium, containing 10% fetal bovine serum (FBS, Gibco). All cells were kept at 37°C in a humidified incubator containing 5% CO_2_.

### Immunohistochemistry

Envision two-step method was adopted for immunohistochemistry. Paraffin-embedded tissues were cut into 4 μm slices, and the dewaxed sections were immersed in antigen repair solution (pH6.0) for microwave repair. Endogenous peroxidases were inactivated with 3% hydrogen peroxide and then occluded with 5% BSA for 30 min. The slices were incubated with primary antibodies for MTHFD1L protein (1:400, Sigma, HPA029041) overnight at 4℃. The secondary antibody (DAKO) was added to the section and incubated at room temperature for 30 minutes. Human kidney tissue was used as positive control, while sections without antibody were used as negative control. Each slide was reviewed by two pathologists, who did not know the patient information. More than 5 high-power fields were observed in each slide, and the mean score of each case was the final score. MTHFD1L staining was considered as positive staining with obvious yellow or brown-yellow particles in the cytoplasm, and the staining result was evaluated by the positive cell rate and staining intensity. According to the proportion of the number of staining cells, the score was 0:0-10%, 1:10% - 25%, 2:25% - 50%, 3:50% -75%, and 4: > 75%. Staining intensity grading score: no staining (-), weak staining (1+), medium staining (2+), and strong staining (3+). And then, the two values were multiplied. Expression intensity was determined: 0-1 was negative, 2-4 was weak positive, 5-8 was moderate positive, and 9-12 was strong positive. Negative and weak positive were considered as negative or low expression, while positive and strong positive were considered as high expression.

### Quantitative RT-PCR

Total RNA was extracted using Trizol reagent (invitrogen). cDNA was synthesized with ReverTra Ace qPCR RT Master Mix with gDNA Remover kit (Toyobo, Code No. FSQ-301). qPCR was performed in a Light Cycler with SYBR Green (Vazyme, China) and the Applied Biosystems 7300 Fluorescent Quantitative PCR system (Applied Biosystems Life Technologies, USA), using the following program: denaturation at 95℃ for 10s, 40 cycles of amplification (95℃ for 10s, 60℃ for 10s ). The following primers were used: Sense, 5'-CTGCCTTCAAGCCGGTTCTT-3', antisense, 5'-TTTCCTGCATCAAGTTGTCGT-3' for MTHFD1L, 5′-CGCGAGAAGATGACCCAGAT-3′ and antisense, 5′-GGGCATACCCCTCGTAGATG-3′ for β-actin.

### Western bolt

Total cell and tissues lysates were extracted by RIPA buffer. Protein concentration were determined using the BCA protein assay kit. Protein were separated by 8% SDS-PAGE gel electrophoresis and transferred to PVDF membranes (Millipore). After blocking, the membrane was incubated with anti-MTHFD1L (1:500, sigma, HPA029041) and rabbit anti-GAPDH (1:5000, Abcam). the ImageJ analysis system was used for density analysis.

### Cell transfection experiment

Knockdown of MTHFD1L was performed using siRNA (Ribobio, China), non-target siRNA served as negative control, the final concentration of 50nM. The transient transfection was performed in DLD-1 and HCT116 cells using the lipofectamine 3000 reagent (Invitrogen) following the manufacture's protocol. The siRNA MTHFD1L sequence: siRNA MTHFD1L #1: GGATGGAGTAACAGACATA, siRNA MTHFD1L #2: GGATGGAGTAACAGACATA. After culture for 48h, the cells were collected for protein extraction or cell function detection.

### MTT cell proliferation assay

After transfection, cells were planked into 96-well plates (2000 cells per well) and 20ul MTT (Invitrogen) was added. After incubation for 4 hours, 200ul dimethyl sulfoxide was added and the absorbance value was measured at 490nm.Repeat for 6, 24, 48, 72 hours.

### Plate colon formation assay

After transfection, the cells were plated on 6-well plates (500 cells per well) and cultured for 10-15 days until the visible clone was seen. The clones were stained with crystal violet and counted.

### Wound healing assay

The cells were plated on 6-well plates, transfected with small interfering RNA, and cultured for 48 hours until the cells were overgrown. The micropipette tip was used to draw straight lines in the vertical petri dish, and then the culture medium of 1%FBS was added after washing with PBS. Pictures were taken under the microscope at 0h, 12h, 24h and 48h, respectively, and the healing area was calculated using ImageJ software. The healing rate was calculated by formula (1-(wound area at the time after wounding)/ (wound area at the time immediately after wounding) × 100).

### Statistical method

All data were analyzed by GraphPad Prism 5.0 and SPSS 19.0 software. Chi-square test was used to analyze the comparison of counting data, and t test was used to analyze the comparison of measuring data. Kaplan-Meier method was used for survival analysis, and univariate Cox and multivariate Cox regression analysis were used for risk factor analysis. All experiments were repeated three times, and P < 0.05 was considered statistically significant.

## Result

### MTHFD1L is highly expressed in CRC

**Figure 2:**
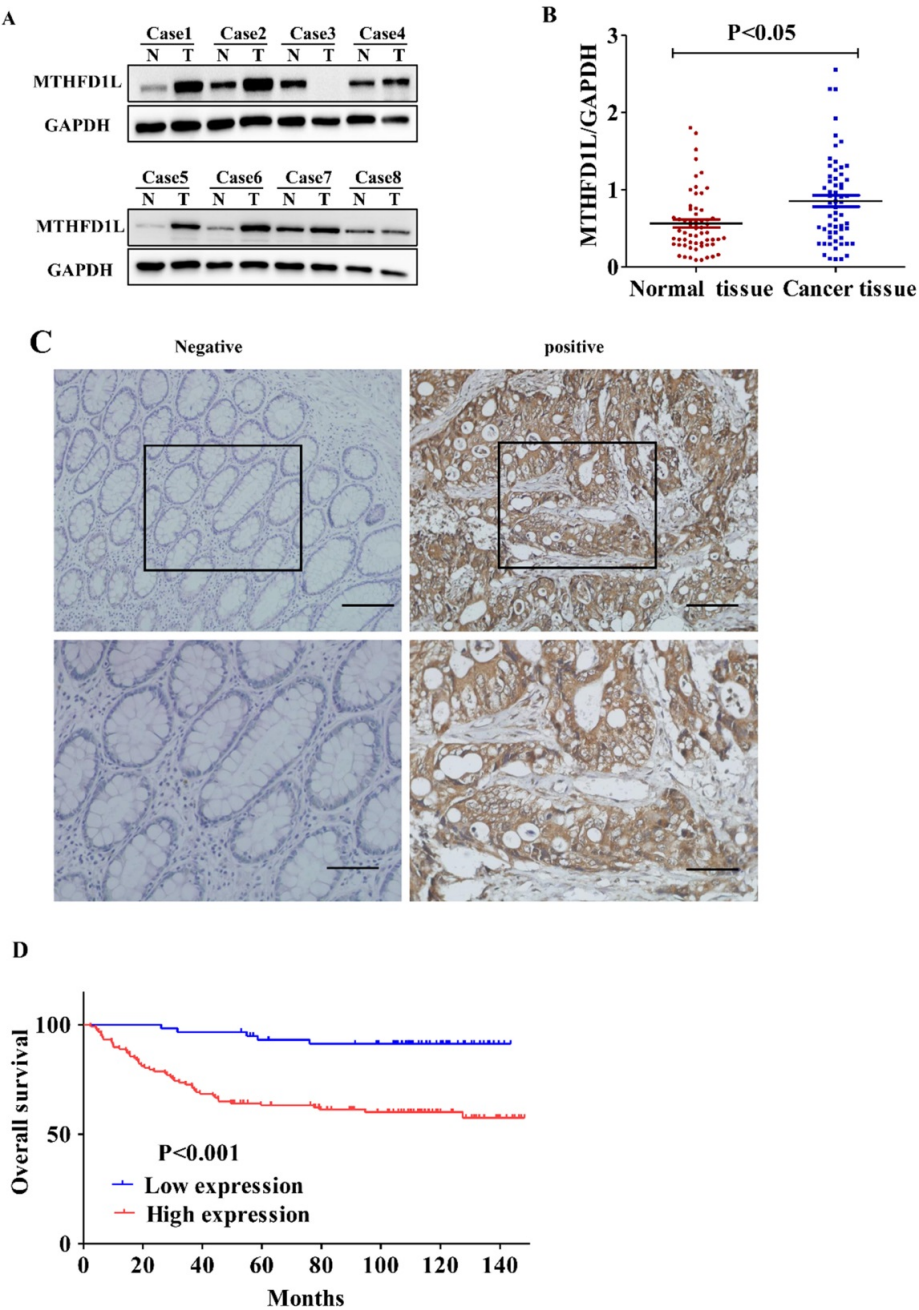
** MTHFD1L is overexpressed in CRC and is associated with poor prognosis. A.** Western blot was used to detect the expression of MTHFD1L in colorectal cancer and para-cancerous tissues. N: The Pairing normal tissue; T: The cancerous tissue. **B.** The relative expression of MTHFD1L protein in CRC tissues (0.9726 ± 0.6942) was higher than that in non-tumor tissues (0.6859±0.4748). 59 case Western blot bands were analyzed using Image J software. The result was the ratio of MTHFD1L to GAPDH. * p < 0.05. **C.** Immunohistochemical staining of MTHFD1L protein on CRC tissues and the corresponding non-tumor tissues. scale bars = 100μm. **D.** Kaplan-Meier analyses of overall survival in 176 CRC patients based on MTHFD1L expression level of CRC tissues.

In an attempt to study the importance of MTHFD1L in CRC, we firstly detected the expression of MTHFD1L between the CRC tissues and adjacent normal tissues in 59 samples by Western Blotting method. The relative quantitative analysis results showed that the expression of MTHFD1L in colorectal tumor tissue were significantly higher than those in the adjacent normal colorectal tissue (P<0.01, Fig. 2, A and B).

In addition, we detected the expression of MTHFD1L in 176 CRC patients by immunohistochemistry methods. Similarly, the results showed that MTHFD1L was highly expressed in CRC tissues (112/176) than in paired normal intestinal mucosal tissues (51/176), (χ^2^=42.516; p <0.01, Fig 2C).

### The relationship between MTHFD1L expression and clinicopathological features in CRC

To analyze the clinical significance of MTHFD1L in CRC, the relationship between MTHFD1L expression and clinicopathological characteristics was performed using chi-square test. The result showed that the expression of MTHFD1L in colon cancer tissues was not significantly correlated with patient's gender, age, tumor size, but positively correlated with the degree of tumor differentiation, TMN classification, tumor invasion depth, lymph node metastasis, and distant metastasis (p<0.05 for all; table 1).

### The relationship between MTHFD1L expression and prognosis in CRC

To analyze the influence of MTHFD1L on the prognosis of CRC patients, the relationship between MTHFD1L and the prognosis of CRC patients was analyzed by K-M Survival and cox regression analysis. K-M Survival analysis showed that the five-year survival rate of CRC patients with high MTHFD1L expression level was significantly dropped than those with low MTHFD1L expression level (61.2±4.5% vs. 93.1±3.3%, P<0.01, Figure 2D). Multivariable Cox analysis indicated that MTHFD1L, Pathology grade, TNM stage, and distant metastasis were independent predictor of poor prognosis in CRC patients ((p<0.05 for all, Table 2). Compared with patients with low MTHFD1L expression, patients with high MTHFD1L expression of colorectal cancer have a higher risk of death (HR=3.927, 95%CI: 1.518-10.16).Survival analysis indicating that MTHFD1L is a potential prognostic biomarker.

### Down-regulating the expression of MTHFD1L reduces the proliferation ability of CRC cells

**Figure 3:**
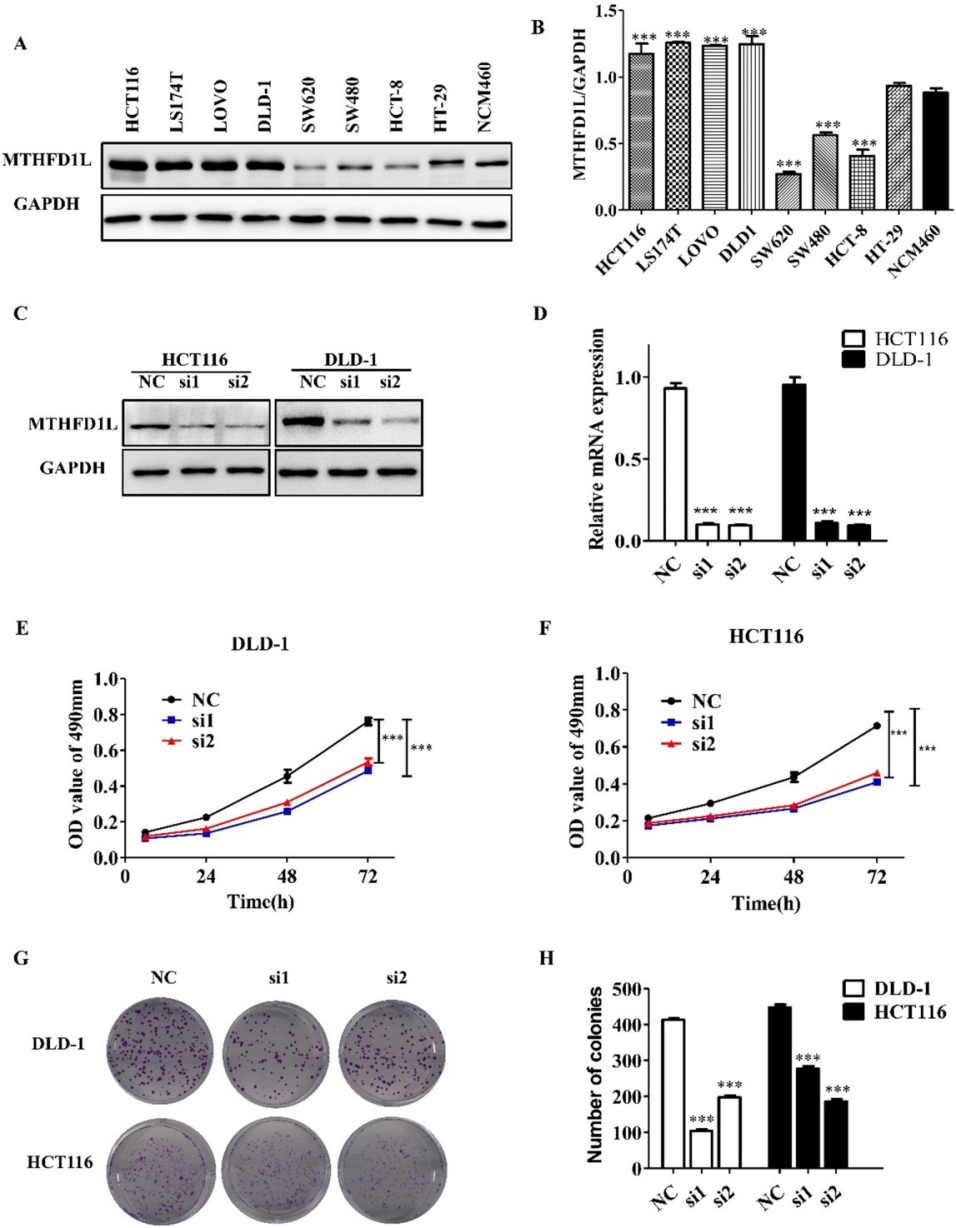
** Down-regulated expression of MTHFD1L reduced the proliferation of colorectal cancer cells. A.** Expression of MTHFD1L in CRC cell lines, and the NCM460 cells were used as the normal control. **B.** The relative expression of MTHFD1L protein in cells, the result was the ratio of MTHFD1L to GAPDH. P<0.05. **C, D.** Knockdown efficiency of MTHFD1L in DLD-1 and HCT116 cell lines in mRNA and protein level. P<0.05. **E, F.** MTT assay showed that down-regulating the expression of MTHFD1L could inhibit the proliferation of colorectal cancer cells. P<0.05. **G.** Down-regulating the expression of MTHFD1L can reduce the clonal formation ability of tumor cells. H. Statistical analysis of the clonal formation ability of low expression MTHFD1L colorectal cancer cells. P<0.05.

The expression of MTHFD1L in CRC cell lines (HCT116, LS174T, LOVO, DLD-1, SW620, SW480, HCT-8, HCT-8, HT-29) was shown in the figure 3A, and the NCM460 cells were used as the normal control. The expression of MTHFD1L in CRC cell lines HCT116, LS174T, LOVO and DLD-1 were significantly higher than NCM460 cells (P<0.05, figure 3, A and B). We selected DLD-1 and HCT116 cells for the further cytological functional experiments.

We down-regulated the expression of MTHFD1L in DLD-1 and HCT116 cells to determine the role of MTHFD1L in cell biology. After the transfection of siMTHFD1L, the mRNA and protein levels of MTHFD1L in DLD-1 and HCT116 cells were significantly decreased (P < 0.05, figure 3, C and D).

MTT assay and plate clonal formation assay were used to detect the effect of reducing MTHFD1L expression on the proliferation of DLD-1 and HCT116 cells. MTT assay showed that, compared with the control group, the growth of cells with low MTHFD1L expression was significantly affected (P < 0.05, figure 3, E and F).

Similarly, the results of plate cloning formation experiment showed that, compared with the control group, the size and number of cell clones with low expression of MTHFD1L were significantly reduced (P < 0.05, figure 3, G and H).

### Down-regulating the expression of MTHFD1L reduces the migration ability of CRC cells

**Figure 4:**
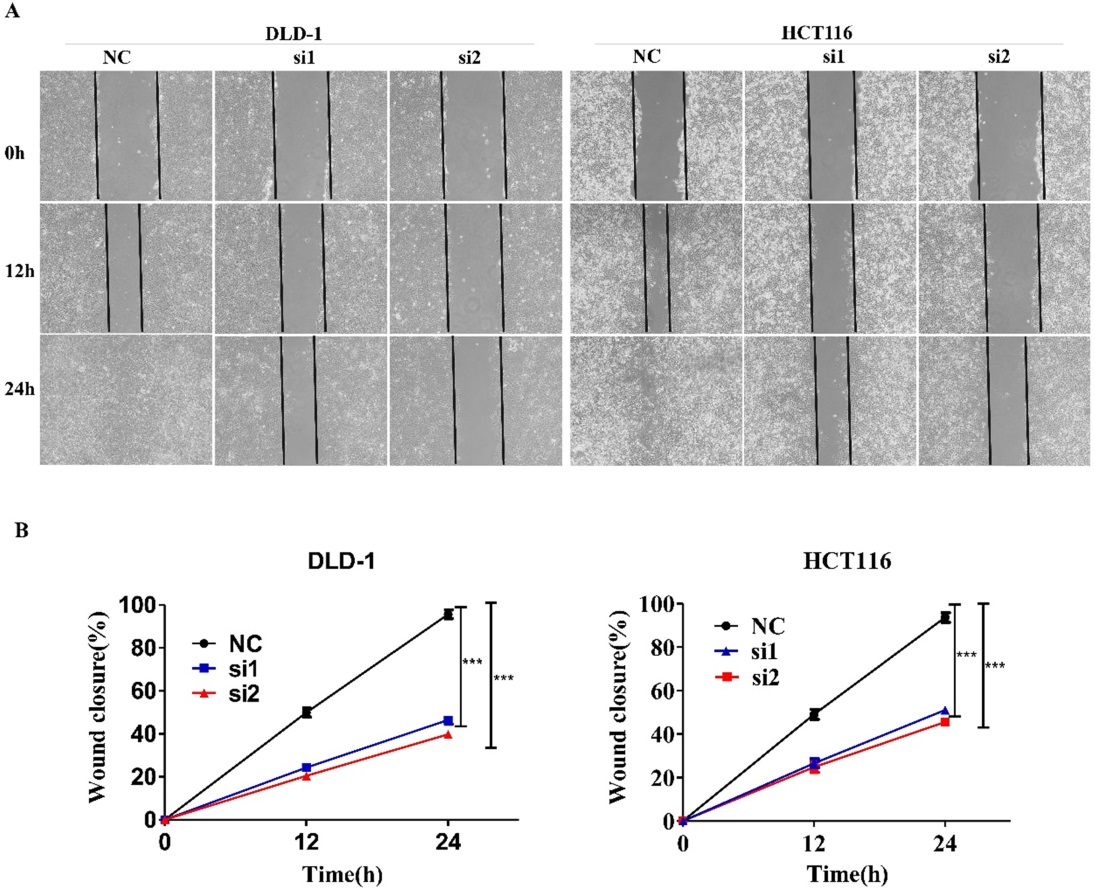
** Down-regulating the expression of MTHFD1L can inhibit the migration ability of colorectal cancer cells *in vitro*. A.** Down-regulating the expression of MTHFD1L can reduce the migration ability of colorectal cancer *cells in vitro*. **B.** Statistical analysis of the migration ability of colorectal cancer cells in vitro after the down-regulation of MTHFD1L expression. *P*<0.05.

We used a wound healing experiment to verify the effect of MTHFD1L on Migration ability of CRC cells. The down-regulation of MTHFD1L has a significant effect on the wound healing of cells, indicating that the down-regulation of MTHFD1L reduces the migration ability of CRC cells. (P < 0.05, figure 4).

## Discussion

Methyltetrahydrofolate dehydrogenase (NADP + dependent)1 like (MTHFD1L) is a single-functional formyltetrahydrofolate synthase encoded by nuclear MTHFD1L on human chromosome 6. MTHFD1L is a key enzyme^16^ involved in the last step of the mitochondrial chamber reaction in the folate cycle, which promotes the production of formate. After entering the cytoplasm, formate can be used as a source of one-carbon unit in the folate cycle^17^, promoting the biosynthesis of nucleotides and methylation and the maintaining the redox state^18^. MTHFD1L plays a crucial role in maintaining folate circulation. Our study showed that MTHFD1L was significantly up-regulated in CRC and could be used as an independent indicator of poor prognosis. Down-regulation of MTHFD1L was associated with decreased proliferation and migration of CRC cells. Summary, these results indicate the key role of MTHFD1L in the progression of CRC, emphasize the prognostic value of MTHFD1L in CRC.

The synthesis of formate provides a one-carbon unit for the folate cycle that provides purines and pyrimidine nucleotides for cell growth^19^. The proliferation of cancer cells also requires a large number of nucleotides, so cancer cells need a large number of one-carbon unit to promote cell proliferation^20^. Further studies showed that the nucleic acid precursors did not enter the carbon cycle, leading to the accumulation of nucleic acid precursors, which is the reason for the inhibition of cell proliferation^20^. Theoretically, decreasing the enzymes involved in folate metabolism can inhibit the proliferation of cancer cells^19^. Studies have shown that down-regulation of MTHFD1L can decrease nucleotide production and thus inhibit the proliferation of esophageal cancer^15^ cells. In this study, downregulation of MTHFD1L also inhibited the proliferation of colorectal cancer cells. Therefore, it is reasonable to assume that MTHFD1L promotes the proliferation of cancer cells by promoting the biosynthesis of nucleotides in the folate cycle.

In cancer cells, increasing the ability to fight oxidative stress is an important step in tumor progression^21^. Therefore, the maintenance of redox homeostasis can mainly offset the increase of reactive oxygen species (ROS) level by improving the antioxidant defense^22^. The maintenance of redox homeostasis is determined by the balance between oxidant and antioxidant levels. The latter is dependent on NADPH production, the basic role of NADPH is to provide electrons so that the redox reaction can tolerate ROS and thus act as an antioxidant defense^22^. Controlling NADPH production increases the ability of cancer cells to tolerate oxidative stress, which promotes tumor development^21^, ^23^, ^24^. The folate cycle is also closely related to NADPH production. MTHFD1L has been shown to increase the ability to resist oxidative stress by promoting NADPH production in hepatocellular cancer^14^. Down-regulating the expression of MTHFD1L made the liver cancer cells sensitive to sorafenib. Inhibition of folate pathway can weaken the anti-oxidative stress ability of tumor cells and promote the metastasis of melanoma^24^. Activation of the Kras^25^ and c-Myc^26^ pathways in colorectal cancer is closely related to folate metabolism. Another key enzyme in the folate cycle, MTHFD2, is transcriptionally activated by c-Myc downstream of Kras, and MTHFD2 promotes colorectal cancer^7^ metastasis by increasing anti-oxidative stress. It is hypothesized that MTHFD1L promotes tumor metastasis by increasing antioxidant stress. Our results showed that MTHFD1L could promote the migration ability of tumor. It can be speculated that MTHFD1L can promote the metastasis of colorectal cancer by increasing the ability of anti-oxidative stress. However, how MTHFD1L controls NADPH homeostasis remains unclear. Next, we will further study the mechanism of MTHFD1L promoting metastasis of colorectal cancer.

In summary, this study indicated that MTHFD1L is highly expressed in colorectal cancer and is associated with poor prognosis. MTHFD1L promotes proliferation and metastasis of colorectal cancer cells, potentially in relation to the biosynthesis of nucleotide and supply of NADPH in one-carbon metabolism. MTHFD1L may be an important prognostic indicator and molecular target for CRC.

## Figures and Tables

**Table 1:** Correlation between MTHFD1L and clinicopathologic parameters.

Variables	n	low expression	high expression	χ^2^ value	*P* value
**Gender**				1.6860	0.1940
Male	102	33	69		
Female	74	31	43		
**Age**				0.0040	0.9493
<50 years	49	18	31		
>=50 years	127	46	81		
**Size**				2.8620	0.0910
<5 cm	98	41	57		
>=5 cm	78	23	55		
**Pathology grade**				7.755	0.0050*
Well+ moderate	133	56	77		
Poor	43	8	35		
**TNM stage**				12.3200	<0.001*
I/II	93	45	48		
III/IV	83	19	64		
**T stage**				8.420	0.0040*
T1/T2	37	21	16		
T3/T4	139	43	96		
**Lymph node metastasis**				11.548	0.0010*
Negative	94	45	49		
Positive	82	19	63		
**Distant metastasis**				fisher	0.0140*
Negative	166	64	102		
Positive	10	0	10		

**Table 2:** Univariate and multivariate analysis for overall survival.

Variable	Univariate analysis					Multivariable analysis			
		95% CI for Exp(B)					95%CI for Exp(B)		
	HR	Lower	Upper	P value		HR	Lower	Upper	P value
MTHFD1L expression: High vs. low	6.701	2.663	16.859	<0.001*		3.927	1.518	10.16	0.005*
Gender: Male vs. female				0.575					
Age(years): ≥50 vs.<50				0.337					
Tumor size(cm): ≥5 vs. <5	2.48	1.417	4.338	0.001*					
Pathology grade: poor vs. (Well+moderate)	5.035	2.914	8.697	<0.001*		4.516	2.552	7.99	<0.001*
TNM stage: Ⅲ+Ⅳ vs.Ⅰ+Ⅱ	4.316	2.301	8.096	<0.001*		2.463	1.265	4.794	0.008*
T stage: T3+T4 vs. T1+T2	5.364	1.671	17.218	0.005*					
Lymphatic metastasis: positive vs negative	3.913	2.118	7.23	<0.001*					
Metastasis: positive vs. negative	9.102	4.32	19.176	<0.001*		5.885	2.638	13.132	<0.001*
